# Preparation and characterization of Fe_3_O_4_@Au-C225 composite targeted nanoparticles for MRI of human glioma

**DOI:** 10.1371/journal.pone.0195703

**Published:** 2018-04-13

**Authors:** Yaoqi Ge, Yuejiao Zhong, Guozhong Ji, Qianling Lu, Xinyu Dai, Zhirui Guo, Peng Zhang, Gang Peng, Kangzhen Zhang, Yuntao Li

**Affiliations:** 1 Second Affiliated Hospital of Nanjing Medical University, Nanjing, Jiangsu Province, China; 2 Jiangsu Cancer Hospital and Jiangsu Institute of Cancer Research, Nanjing, Jiangsu Province, China; Brandeis University, UNITED STATES

## Abstract

**Objective:**

To study the characterization of Fe_3_O_4_@Au-C225 composite targeted MNPs.

**Methods:**

Fe_3_O_4_@Au-C225 was prepared by the absorption method. The immunosorbent assay was used to evaluate its absorption efficiency at C225 Fc. ZETA SIZER3000 laser particle size analyzer, ultraviolet photometer and its characteristics were analyzed by VSM. the targeting effect of Fe_3_O_4_@Au-C225 composite targeted MNPs on U251 cells *in vitro* were detected by 7.0 Tesla Micro-MR; and subcutaneous transplanted human glioma in nude mice were performed the targeting effect in vivo after tail vein injection of Fe_3_O_4_@Au-C225 composite targeted MNPs by MRI.

**Results:**

The self-prepared Fe_3_O_4_@Au composite MNPs can adsorb C225 with high efficiency of adsorption so that Fe_3_O_4_@Au-C225 composite targeted MNPs were prepared successfully. Fe_3_O_4_@Au-C225 composite targeted MNPs favorably targeted human glioma cell line U251 *in vitro*; Fe_3_O_4_@Au-C225 composite targeted MNPs have good targeting ability to xenografted glioma on nude mice *in vivo*, and can be traced by MRI.

**Conclusion:**

The Fe_3_O_4_@Au-C225 composite targeted MNPs have the potential to be used as a tracer for glioma *in vivo*.

## Introduction

The primary malignant central nervous system tumor is among 1.49% of all cancer and the rate of disability and mortality is considerably high [1, 2]. Most of these tumors originate from the gliocyte, which is commonly abbreviated to glioma. Despite advances in diagnosis and treatment for it, the prognosis is still disappointing. Therefore, there exists an urgent need for better and more effective treatment. Over the past few decades, neurologist and oncologist have dedicated to understanding the mechanisms of glioma formation and developing methods to stabilize, reduce or even eliminate the tumor.

The nanometer science and technology is developing rapidly nowadays. It motivates more and more interdisciplinary integration between various subjects to create new fields of research and growing branches of the subject. The nanoscale device, with merely 1~100nm in its length, can enter and leave human cells freely. It has the advantages of less volume, better biocompatibility, and superior targeting ability to specific tissus/cells compared to traditional tracers. In our previous study, we synthesized Fe_3_O_4_@Au composite magnetic nanoparticles (MNPs) with crystal growth to optimize the preparation process and its biocompatibility [3]. Gold has a strong capacity in absorbing protein molecules, therefore provides a microenvironment which is similar to the biological molecular ontology environment. Thus, using gold as active core–shell for constructing composited MNPs helps to maintain the reactivity of biological component well [4]. Furthermore, it can enhance the stability of MNPs to improve the binding ability between MNPs and targeted molecules, resulting in stable coupling between MNPs and antibody [5]. Meanwhile, gold can mediate NIR thermotherapy as a sensitizer of light for hyperthermia depended electron-dense, dielectric property, high adsorption cross section and high Light-thermal conversion efficiency [6]. Thus, it can be used for fixing EGFR monoclonal antibody (McAb) cetuximab (C225), constructing new nanocomposite which gathers molecular targeted therapy, MRI, MFH and NIR thermotherapy by preparing Fe_3_O_4_@Au composite MNPs. The preparation and application of Fe_3_O_4_@Au composite MNPs have been reported before the present study, but the research of it used as a carrier to fix commercially available EGFR McAb-C225 for anti-glioma has not been conducted before. This article uses the method of MRI to evaluate the targeting ability of the self-prepared Fe_3_O_4_@Au-C225 composite targeted MNPs *in vivo* and *in vitro*.

## Materials and methods

### Main apparatus and reagents

The ultrasonic cleaner used was a CQ50 model (Ultrasonic Instrument Factory, Shanghai). The water-bathing constant temperature vibrator used was an SHZ-22 model (Medical Apparatus Factory, Taicang, Jiangsu). The automatic steam generator used was a ZFQ-B model (Xinhua Medical Apparatus Company, Shandong). The constant current electrophoresis apparatus was purchased from Liuyi Instrument Factory (Beijing). The ultraviolet detector was purchased from Tian Neng Company (Shanghai). The microplate reader used was a Multiskan MK3-353 model (USA). The double-door and dual-temperature refrigerator was purchased from Haier (China). The vacuum drying oven used was a 668 model (Dongtai Electrical Equipment Factory, Jiangsu). The vibrating sample magnetometer (VSM) used was a PPMS-9 model (Quantum Design, USA). A 752 prismatic ultraviolet-visible (UV-vis) spectrophotometer was purchased from Shanghai Precision Scientific Instrument Co, Ltd, (Shanghai, China). The 7.0Tesla Micro- MR was purchased from PharmaScan (Bruker). The Inverted microscope was purchased from Nikon (Japan). Cetuximab Solution for Infusion (C225) was purchased from Merck (Germany). Fe3O4@Au composite MNPs were prepared at the Department of Imaging and Nuclear Medicine, School of Medicine, Southeast University. All reagents were of analytical grade.

### Cells and animals

Cell line U-251(human glioma cells) was purchased from Shanghai Institute of Cell Biology (Shanghai, China). Balb/c nu/nu nude mice, age-matched (5~7 weeks of age) and weight matched (18–22 g), were provided by the center of Slac Laboratory Animal of Chinese Academy of Sciences (Shanghai, China). All mice experiments were evaluated and approved by the Animal and Ethics Review Committee of Second Affiliated Hospital of Nanjing Medical University (Nanjing, Jiangsu Province, China). All mice were maintained in a pathogen-free, air-conditioned environment at 24°C ± 2°C with a standard 12-hour light/12-hour dark cycle.

### Preparation and characterization of Fe_3_O_4_@Au-C225 composite targeted MNPs

#### Adsorption immune response

2.5ml Fe_3_O_4_@Au composite MNPs solution (1g/L) was taken, the PH of nano-gold solution be adjusted to 9.0 with HCL solution (0.1mol/L), 20uL C225 (5 mg/mL) was added, the solution was concussed in air to fully mixed react with speed at 200 rpm and 37°C for 2h. Then 100uL BSA solution (mass fraction is 1%) was incrementally added to reacted for 30min and magnetic separated for 20min. The supernatant was removed carefully and the sediment was dissolved in 1% BSA solution (1mL). The final solutiion was stored at 4°C after shaking.

#### The test of adsorption effect

The 100ul Fe_3_O_4_@Au-C225 solution was added into 1ml Goat Anti-Human Fc-HRP solution (500, 1000, 2000, 4000, 8000, 16000 times dilute solution of HRP-labeled antibody, pH = 7.4). The reaction was carried out in the water bath at 37°C for 60 minutes. Then the solution was magnetic separated at 4°C for 60 minutes, and the supernatant solution (100ul) was reacted in 100ul substrate solution of o-phenylene diamine(OPD) from light for 10 minutes. The reaction was stopped with 50uL Sulfuric Acid (2mol/L). Then the optical density (OD) value was measured at 492 nm on a microplate reader. In the control experiment, the surface active spot of Fe_3_O_4_@Au was completely enclosed by BSA, then it was added into C225 solution to obtain the mixed solution of Fe_3_O_4_@Au-BSA and C225. The 100ul mixed solution was added into 1ml Goat Anti-Human Fc-HRP solution (500, 1000, 2000, 4000, 8000, 16000 times dilute solution of HRP-labeled antibody, pH = 7.4) as well, following the same steps mentioned above.

#### Particle size and Zeta potential test of Fe_3_O_4_@Au-C225 composite targeted MNPs

The Fe_3_O_4_@Au-C225 composite targeted MNPs solution prepared above was placed in a cuvette. A ZETA SIZER3000 laser particle size analyzer and a dynamic light scattering software were used to detect their average diameter and surface potential. The morphology and size of the Fe_3_O_4_@Au-C225 composite targeted MNSs were investigated by TEM imaging.

#### Optical properties test of Fe_3_O_4_@Au-C225 composite targeted MNPs

The Fe_3_O_4_@Au-C225 composite targeted MNPs solution and Fe_3_O_4_@Au composite MNPs solution prepared above were placed in individual cuvette. The UV-vis spectra were acquired with a 752 prismatic UV-vis spectrophotometer.

#### Magnetic properties test of Fe_3_O_4_@Au-C225 composite targeted MNPs

The magnetization *in vitro* of Fe_3_O_4_@Au-C225 composite targeted MNPs freeze drying powder was measured by a vibrating sample magnetometer (VSM) in the magnetic field range of -5000Oe~+5000 Oe at 300K.

### Targeting ability evaluation of Fe_3_O_4_@Au-C225 composite targeted MNPs *in vitro*

#### Glioma cell culture

U251 human glioma cell line was seeded in 10% fetal calf serum high glucose DMED medium. At 37°C, 5% CO2 conditions in the incubator, passage every 2~3 days, to make the cells adhere to the surface.

#### Observation of U251 cells labeled by Fe_3_O_4_@Au-C225 composite targeted MNPs *in vitro*

U251 cells in logarithmic growth phase were seeded in 4 holes of a 6-well plate. About 1 x 106 cells were in each well, the cells in 3 of the 4 holes were labeled after 24h for adhere. The concentration of Fe_3_O_4_@Au was 0.01mg/ml, 0.05mg/ml, 0.1mg/ml and another unlabeled one. After incubation for 24h, the best concentration of probe labeled was observed with an inverted microscope. This concentration was used in all follow-up experiments.

#### MRI of labeled U251 cells *in vitro*

U251 cells in logarithmic growth phase were seeded in a 6-well plate at a concentration of 1 x 106 cell/well and divided into group a, b and c after 24 h. Then the cells were incubated for 24 with the Fe_3_O_4_@Au-C225 composite targeted MNPs (group a), Fe_3_O_4_@Au (group b) and Fe_3_O_4_@Au-C225composite targeted MNPs + C225 (40μg/mL) (group c). 0.25% trypsin was used to digest the cells, after collection and centrifugation, it was resuspended in Eppendorf tube within 0.5ml 1% agarose, and another Eppendorf tube was used as control. 7.0 Tesla Micro-MR (Bruker, PharmaScan 7.0), body coil with an inner diameter of 3 cm and T2-weighted spin-echo images was used for MRI, detailed parameters go as follows: Field of view (FOV) was 5cm x 5cm; layer thickness was 1mm; matrix was 256 x 256; TR was 2000 ms, TE was 36 ms.

### Targeting ability evaluation *in vivo* of Fe_3_O_4_@Au-C225 composite targeted MNPs

#### Transplanted model of human glioma in nude mice

U251 cells in logarithmic growth phase were digested by 0.25% trypsin and centrifugated. Then, the cells dissolved in 0.9% saline with a concentration of 2 x 106 cells were injected subcutaneously into the right lower extremity of nude mice. 0.15ml solution and 1cm from injection point to the needle point was carried out for injection. The tumors started to grow in inoculation site after 2–3 weeks. It was used in experiment when the diameter of tumor up to 0.5 cm after 4 weeks.

#### MRI of Fe_3_O_4_@Au-C225 composite MNPs targeted glioma

The mice were randomly divided into group a, b and c, with six mice in each group. Fe_3_O_4_@Au-C225 composite targeted MNPs dissolved in 0.9% saline with a concentration of 100 mg/mL was injected into tail vein of the mice at a dosages of 10mg/kg in group a, the same volume of Fe_3_O_4_@Au composite MNPs was used in group b and Fe_3_O_4_@Au-C225 composite targeted MNPs + C225(40μg/ mL) was used in group c. The changes of MR signal intensity of tumorous were observed by 7.0 Tesla MR Images before and 2h, 8h, 24h and 48h after injection. Imaging sequences include SE-T1WI, SE-T2WI and GRE-T2*WI. The extent and scope of changes and the change over time of tumor signal were evaluated by quantitative analysis.

## Results

### Preparation and identification of Fe_3_O_4_@Au-C225 composite MNPs

#### Adsorption immune response and the test of adsorption effect

The adsorption effect of Fe_3_O_4_@Au-C225 composite MNPs on C225 was tested by the dilute solution of the HRP-labeled antibody with 500, 1000, 2000, 4000, 8000, 16000 times, the OD value was tested from supernate. The mixed solution of Fe_3_O_4_@Au-BSA and C225 was used as controls. (Table 1).

**Table 1:** Adsorption effect of Fe_3_O_4_@Au-C225 to antibody Fc.

Dilution Factor	Fe_3_O_4_@Au-C225	Fe_3_O_4_@Au-BSA, C225	Adsorption Rate (%)
500	0.782	0.813	96.2
1000	0.736	0.778	94.6
2000	0.734	0.789	93.0
4000	0.606	0.654	92.7
8000	0.534	0.582	91.8
16000	0.346	0.378	91.5

#### The size distribution and Zeta potential of Fe_3_O_4_@Au-C225 composite targeted MNPs

**Fig 1:**
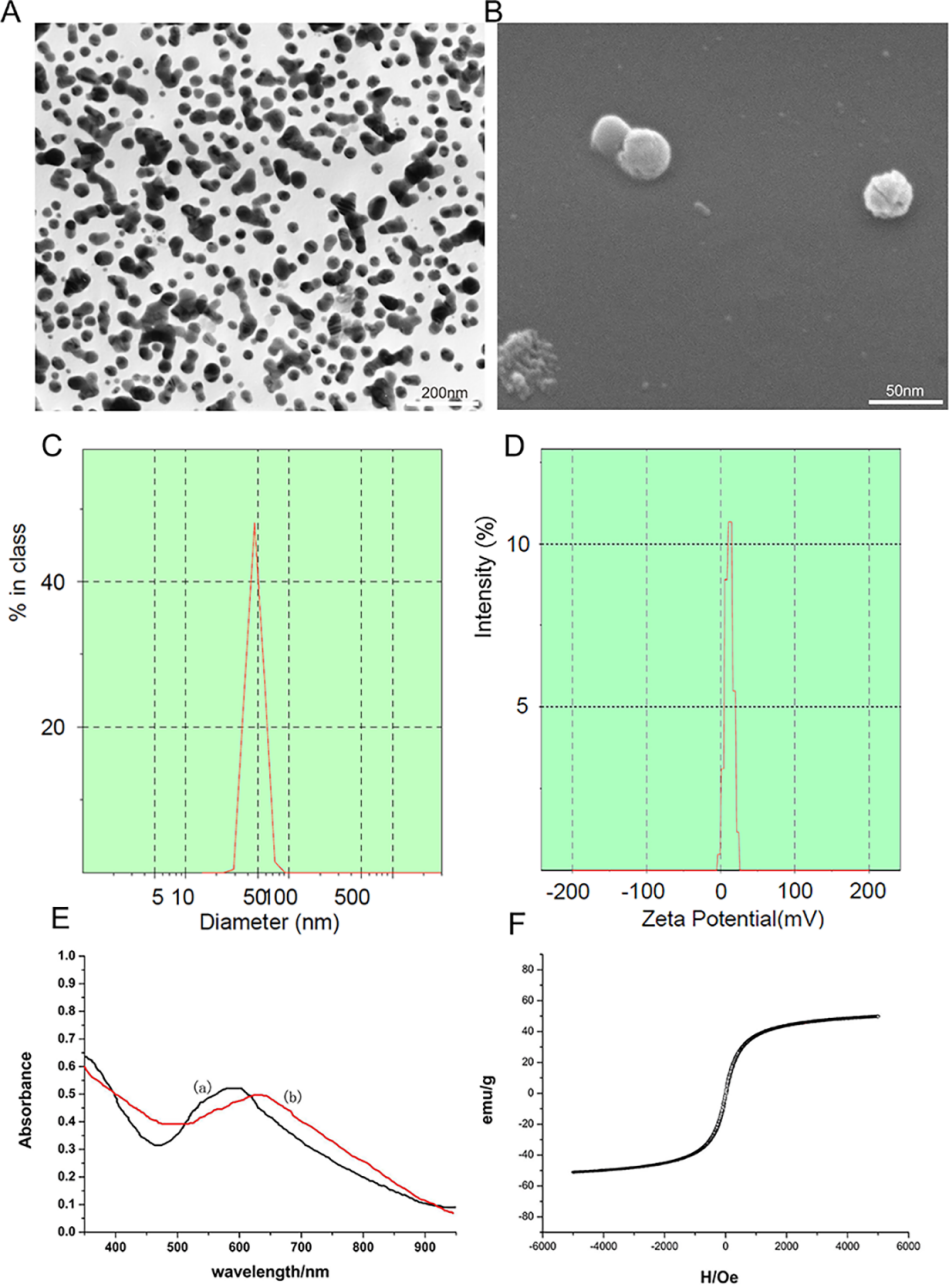
Transmission electron microscopy image of Fe3O4@Au composite MNPs (A); scanning electron microscopy image of Fe3O4@Au composite MNPs (B); The average diameter of Fe3O4@Au-C225 composite targeted MNPs (C); The zeta potential of Fe3O4@Au-C225 composite targeted MNPs (D); The UV–vis absorption spectra of Fe3O4@Au-C225 composite targeted MNPs (curve b) and Fe3O4@Au composite MNPs (curve a)(E); The hysteresis loops of Fe3O4@Au-C225 composite targeted MNPSs(F).

The self-prepared Fe3O4@Au-C225 composite MNPs were approximately spherical and uniform in size as observed by TEM and SEM (Fig 1A and 1B).The average diameter of Fe_3_O_4_@Au-C225 composite targeted MNPs was 46nm, showed unimodal distribution and narrow size distributed (Fig 1C). The Zeta potential value of Fe_3_O_4_@Au-C225 composite targeted MNPs was 11.1±1.8 mV in a neutral environment of pH = 7.4 (Fig 1D).

#### Optical properties of Fe_3_O_4_@Au-C225 composite targeted MNPs

The absorption peaks of Fe_3_O_4_@Au solution was 612nm and Fe_3_O_4_@Au-C225 solution was 630nm. Not only the absorption peaks of Fe_3_O_4_@Au-C225 was decrease but also appeared obvious red shift at the same dilution multiple. This indicated that C225 was well adsorbed on the surface of gold nanoparticle. (Fig 1E curve b)

#### Magnetic properties of Fe_3_O_4_@Au-C225 composite targeted MNPs

The magnetic hysteresis loop showed that the magnetism increased along with the increase of the intensity of externally applied magnetic field of Fe_3_O_4_@Au-C225 composite targeted MNPs at 300k, but they tend to be saturated eventually. The magnetization intensity was tended to 0 when the externally applied magnetic field was dropped gradually to 0, and tend to be saturated in reverse by applied a magnetic field reversed. The magnetic hysteresis loop was closed to coincidence S-curve and showed good superparamagnetism and low remanence and coercivity. The saturation magnetization (Ms) of Fe_3_O_4_@Au-C225 composite targeted MNPs was 51.2emu/g, it was closed to the Ms of Fe_3_O_4_@Au-C225 composite MNP which was 51.8emu/g (Fig 1F).

### Targeting ability evaluation of Fe_3_O_4_@Au-C225 composite targeted MNPs *in vitro*

#### Morphology observation of tumour cell and concentration exploration of labelled cells

**Fig 2:**
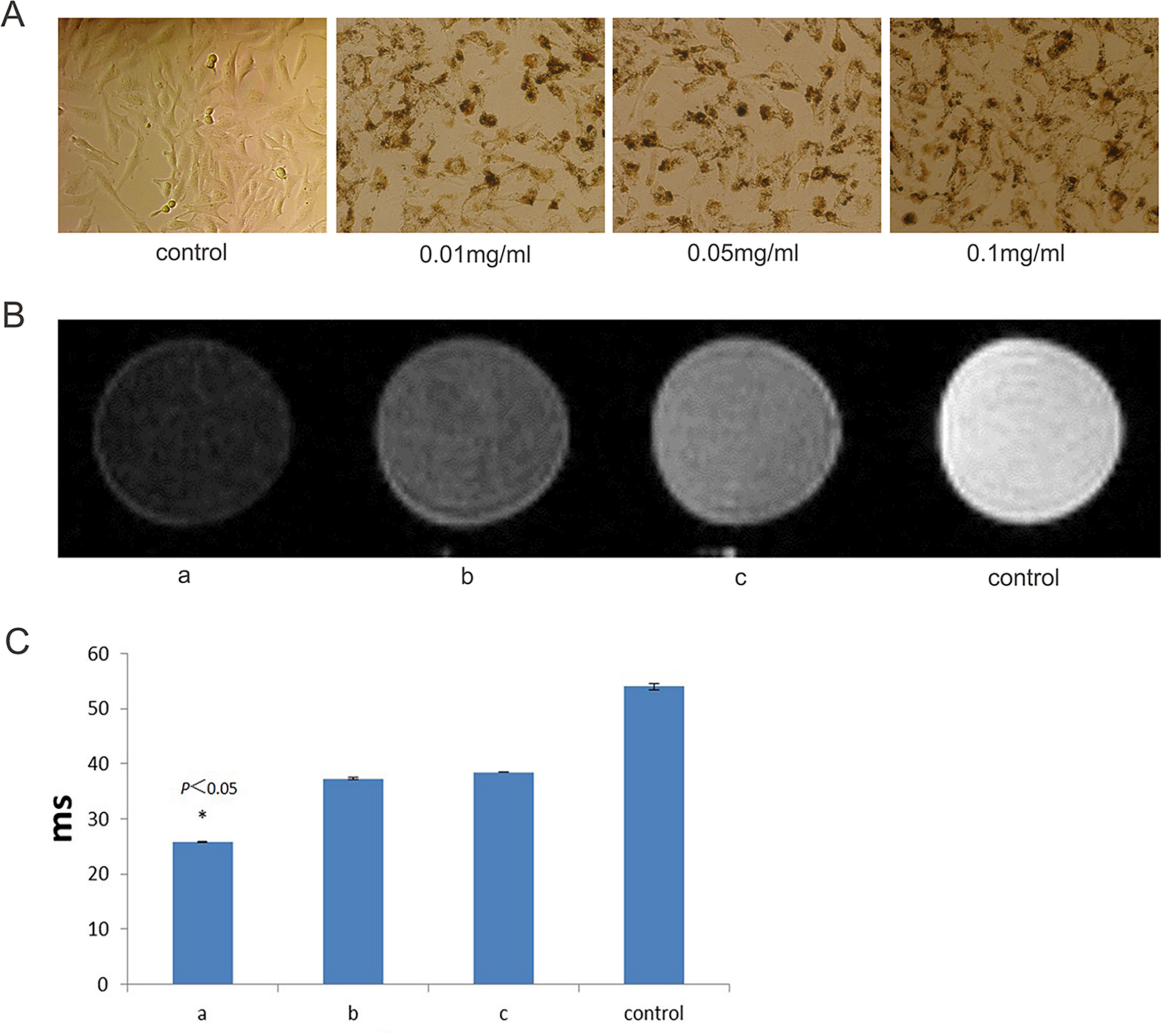
The U251 cells labeled by Fe3O4@Au-C225 composite targeted MNPs at the concentration of 0.01mg/mL, 0.05mg/mL and 0.1mg/mL (A); The MRI of the U251 cells labeled by Fe3O4@Au-C225 composite targeted MNPs (a), Fe3O4@Au composite MNPs (b), both Fe3O4@Au-C225 composite targeted MNPs and C225 (c)(B); The T2WI relaxation time of the U251 cells labeled by Fe3O4@Au-C225 composite targeted MNPs (a), Fe3O4@Au composite MNPs(b), both Fe3O4@Au-C225 composite targeted MNPs and C225(c)(*compared with the control group, P<0.05)(C).

Under reverse microscopy, the U251 cells were homogeneous, transparent and irregularly flat polygonal. They were arranged in single layer tightly, and the size and shape showed diversity. The spindle cell and polygonal cells which have relatively large nucleus were more commonly existed. They have abundant cytoplasm, obvious cellular atypia and different-sized nucleus. And the nucleus showed a large percentage and an increase hyperchromasia and nucleoli. The U251cells were labeled by Fe_3_O_4_@Au-C225 composite targeted MNPs at the concentration of Fe_3_O_4_@Au of 0.01mg/ml and 0.05mg/ml and 0.1mg/ml (Fig 2A). It was showed that, the internalization of Fe_3_O_4_@Au-C225 composite targeted MNPs by U251 cells was less at 0.01mg/ml, and the background was clear and there was no extracellular particle aggregation; the internalization was more, the background was clear and there was no extracellular particle aggregation at 0.05mg/ml; the internalization was more, but there was more extracellular particle aggregation at 0.1mg/ml. So we chose 0.05mg/ml as the label concentration for further study.

#### MRI *in vitro*

The MRI *in vitro* showed that, the signal intensity in T2WI was obviously reduced contrasted with control group after the U251 cells were incubated with Fe_3_O_4_@Au-C225 composite targeted MNPs (group a, targeting group). Then the signal intensity in T2WI was no obviously reduced after the U251 cells were incubated with Fe_3_O_4_@Au composite MNPs (group b, non-targeting group). And the signal intensity in T2WI was also no obviously reduced after the U251 cells were incubated with Fe_3_O_4_@Au-C225 composite targeted MNPs + C225 (group c, targeting inhibition group) (Fig 2B and 2C).

### Targeting ability evaluation in vivo of Fe_3_O_4_@Au-C225 composite targeted MNPs

#### Transplanted model of human glioma in nude mice

The tumor forming rate of U251cells was about 70%.

#### MRI of Fe_3_O_4_@Au-C225 composite targeted MNPs targeted glioma in nude mice

**Fig 3:**
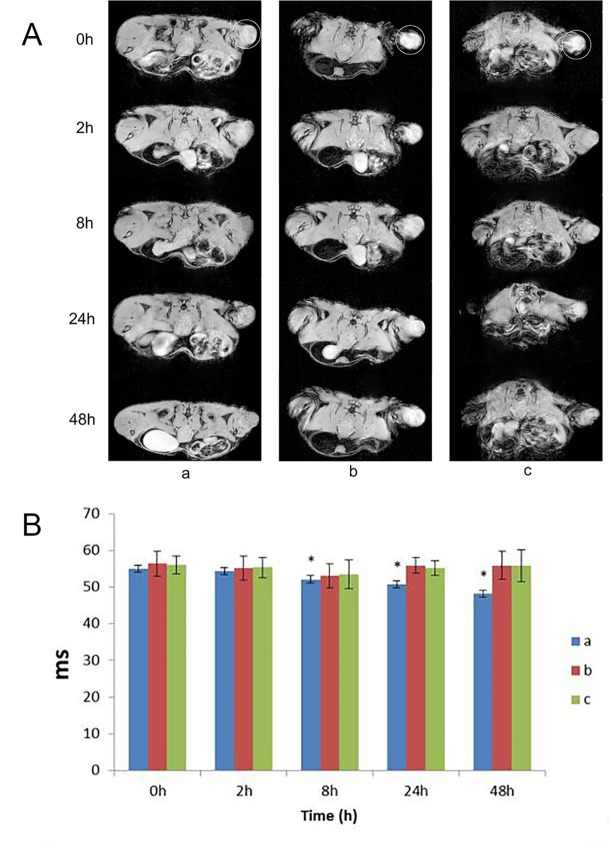
The MRI T2*WI of the human transplanted glioma in mice labeled by Fe3O4@Au-C225 composite targeted MNPs(a), Fe3O4@Au composite MNPs(b), both Fe3O4@Au-C225 composite targeted MNPs and C225(c) (circle for the glioma)(A); The T2*WI relaxation time of the human glioma transplanted in nude mice labeled by Fe3O4@Au-C225 composite targeted MNPs(a), Fe3O4@Au composite MNPs(b), both Fe3O4@Au-C225 composite targeted MNPs and C225(c) at different time points (*compared with the 0h time point, P<0.05)(B).

The MRI *in vivo* showed that, the signal intensity and the change rate of tumor were significantly reduced at different time points both in T2WI and T2*WI after injecting Fe3O4@Au-C225 composite targeted MNPs, which has statistics difference. Although the signal showed slight decrease in group b and group c at 2 h and 8 h after injection, the decrease was less pronounced than group a, and took a shorter duration. The signal intensity of group a was still maintained at a lower level at 24h after injection, and continued to decrease with time. By contrast, the signal intensity of group b and group c were recovered to plain scan level at 24h after injection (Fig 3A and 3B).

## Discussion

### Preparation and identification of Fe_3_O_4_@Au-C225 composite targeted MNPs

#### Prepared scheme of Fe_3_O_4_@Au-C225 composite targeted MNPs

Recently, the molecular pathology study of glioma showed that the overexpression of EGFR is always present in patients with malignant glioma [7]. The overexpression of EGFR is related to poor prognosis, a shortened disease-free period and drug resistance [8–10]. EGFR is the first member of the erbB tyrosine kinase family, encoded by the proto oncogene, to be found. The major endogenic ligand of EGFR are EGF and transforming growth factor α (TGF-α). They play an important role in the proliferation of normal and malignant epithelial cells. The EGFR-dependent downstream signaling transduction pathway mainly includes RAS/MAPK, PI3K/Akt, STAT and PLCγ [11]. These pathways can promote cell proliferation, inhibit apoptosis and differentiation, increase the expression level of the VEGF, and promote the invasion and distant metastasis of tumors [12–14]. Thus, EGFR is undoubtedly a preferred key gene and target site in terms of treatment of glioma.

C225 is a human-mouse chimeric IgG1 monoclonal antibody. It competes with EGF and TGF-α to bind with the ligand in the extracellular domain of EGFR. It inhibits the autophosphorylation of EGFR, blocking downstream signaling transduction pathways, and inhibits signal transmission of cell mitosis, which is activated by growth factor and prevents tumor cell proliferation [15]. In addition, the combination of C225 and EGFR can also induce the engagement of receptors in dimerization, internalization and down-regulation [16]. C225 can inhibit the progression of the cell cycle from the G1 stage to S stage, induce apoptosis of tumor cells, inhibit tumor angiogenesis and inhibit the invasion and metastasis of tumors by a variety of activators [17]. Increased antitumor effects of cytotoxic drugs and restored sensitivity of resistant cells were observed when C225 was combined with antitumor drug [18]. C225 can prevent radiation injuries of tumor cells.

**Fig 4:**
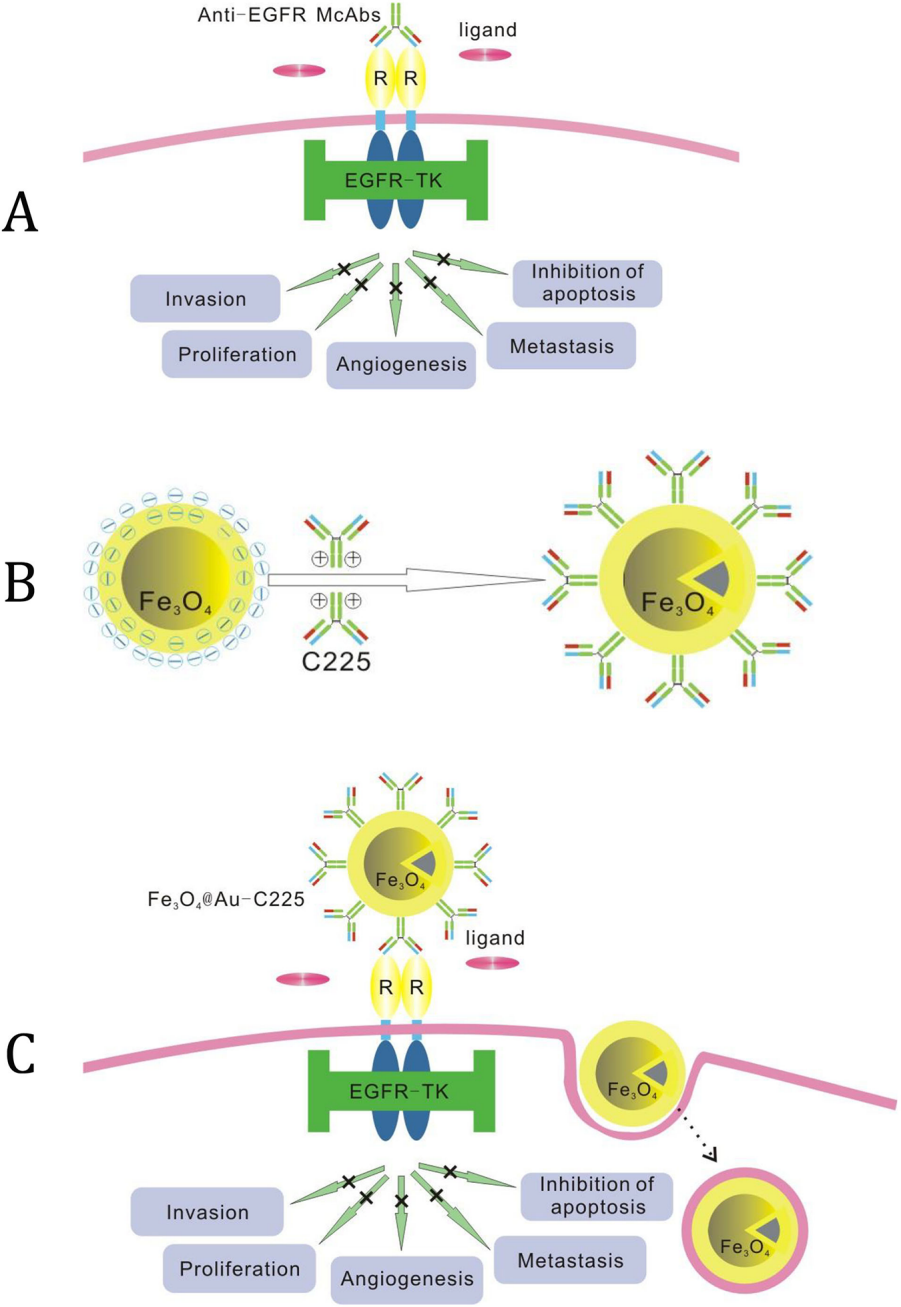
Schematic illustration of McAb-C225 blocking EGFR (A); Schematic illustration of C225 McAb adsorbed by Fe3O4@Au composite MNPs (B); Schematic illustration of McAb-C225 mediated endocytosis for Fe3O4@Au-C225 composite targeted MNPs (C).

C225 is currently the first drug approved for use on head and neck tumors by the FDA [19]. This is due to its excellent ability to target EGFR and its synergistic effect with chemotherapy and radiation. In order to explore whether or not it has a synergistic effect with the thermotherapy of glioma, EGFR-targeted composite MNPs are constructed using MNPs as a carrier and C225 as a target molecule, where C225 is fixed on the MNPs surfaces. In our previous research, the Fe_3_O_4_@Au composite MNPs with a good biocompatibility was prepared [3]. Additionally, using the absorptive abilities of antibodies on gold surfaces, Fe_3_O_4_@Au-C225 composite MNPs was successfully made through ionic and hydrophobic interaction. (Fig 4A)

#### Adsorption of C225 and the test of adsorption effect

Presently, common methods used for antibody fixation include adsorption, entrapment, crosslinking and covalent binding. Adsorption is a method used to fix antibodies on solid surface through physical adsorption [20]. The method generally involves the immersion of a solid state transducer in a solution containing antibodies, after the solid surface has undergone appropriate treatment or modification, or consists of dropping the solution on the solid surface for adsorption via polar bonds, hydrogen bonding, hydrophobic interactions and interactions between electrons. The adsorption methods commonly includes: (1) Nano particle adsorption: the absorption between gold nanoparticle and antibody to fix the antibodies on the surface of gold nanoparticles [21]. (2) Self-assembly absorption: the characteristic interaction between the sulfenyl of antibody itself, and gold to fix onto the surface of gold [22–23].

The method of adsorption to fix C225 on the Au shell of Fe_3_O_4_@ Au composite MNPs was adopted to maintain maximum antibody activity after fixation. Other methods result in reactions that are difficult to control, large losses of antibody activity, or be complicated to operate with high costs. According to the colloidal gold labelled technique, the adsorption of gold nanoparticles onto a protein mainly depends on pH levels. They can easily form a solid combination under the conditions close to the protein isoelectric point or in alkaline conditions. The combination between gold nanoparticles and IgG was the most stable at a pH of 9.0. The combination was currently considered of the adsorption by electrostatic interaction between negative charge on the surface of gold and positive charge group of antibody and form a solid combination by stable chemical bond such as Au-S [24–27]. The Fe_3_O_4_@Au composite MNPs prepared was an average size of 35 nm with a negative charged on the surface (-23.2±1.8 mV), and was able to immobilize IgG monoclonal antibody C225 effectively. (Fig 4B)

The optimum amount of C225 absorbed was based on the salts that affect the adsorption of gold colloids on antibodies to make the sol coagulation. The results showed that, Fe_3_O_4_@Au composite MNPs solution can be stable with 30–60μg/ml C225, which means the proportion of C225 to Fe_3_O_4_@Au composite MNPs was appropriate under this mass concentration range. 40μg/ml of C225 solution was chosen for adsorption to obtain the best adsorption effect and to save cost. BSA is widely used in biochemical experiments. It was added in the enzyme—cutting reaction buffer to protect the enzyme by improve the concentration of protein in the solution. That can prevent the decomposition of enzymes and non-specific adsorption, which can reduce the degeneration caused by adverse environmental factors such as heating, surface tension and chemical factors of some enzymes.

The specific action of antibody and other biomolecules was due to their specific functional areas, such as the Fab-terminal of antibody recognizing the corresponding antigen, but Fc-terminal not having this ability [28]. In order to maintain the bioactivity of antibody at a maximum, the fixed adsorption and other modifications of the antibody can only be carried out at the Fc-terminal. So, the Fc-terminal of the antibody is clearly crucial to the adsorption condition of Fe_3_O_4_@Au composite MNPs. The adsorption condition of Fe_3_O_4_@Au composite MNPs and C225 was studied by Eenzyme-Llinked Iimmunoadsorbent Aassay (ELISA), which is common. The adsorption efficiency of Fe_3_O_4_@Au-C225 composite MNPs on Fc-end of C225 can be obtained when there is complete binding capacity of Goat Anti-Human Fc-HRP to exposed Fc-end of C225. The Fe_3_O_4_@Au composite MNPs can not only be formed with antibodies but also from precipitation by magnetic separation. The Fe_3_O_4_@Au was considered to adsorb either the Fc-end or Fab-end after C225 was added into Fe_3_O_4_@Au solution. The Fc-end of C225 cannot combine with Goat Anti-Human Fc-HRP after it was adsorbed by Fe_3_O_4_@Au. Then the Fc-end of C225 without combined with Fe3O4@Au can be combined with Goat Anti-Human Fc-HRP. After that, it must lead to a decrease of Goat Anti-Human Fc-HRP in supernatant by magnetic separation. If Fc-end of C225 was completely combined with Fe_3_O_4_@Au, the quantity of Goat Anti-Human Fc-HRP would not decrease after centrifugation. Thus, the Fe_3_O_4_@Au composite MNPs-C225- Goat Anti-Human IgG complex can be separated from a solution by magnetic separation after the second antibody of Goat Anti-Human IgG Fc-HRP is added into a solution of Fe_3_O_4_@Au composite MNPs adsorbed C225. Then, the enzyme labeled antibody, which is not combined with C225, is reserved in the solution. It can result in coloration after the addition of a substrate. The OD value can be obtained via adding enzyme substrate for coloration. If same concentrations of C225 solution and Goat Anti-Human IgG are reacted at the same time, the OD value can be measured by coloration, using the addition of substrates. Then the adsorption rate of Fe_3_O_4_@Au composite MNPs on the Fc-terminal of C225 can be obtained from the difference between two OD values. The mixed solution was used as a control to calculate the adsorption efficiency to avoid the affection of coloration by Fe_3_O_4_@Au in experiment. The results showed that the absorption efficiency of Fe_3_O_4_@Au composite MNPs on the Fc-terminal of C225 was above 90%, which meant that the adsorption effect of Fe_3_O_4_@Au composite MNPs on C225 was relatively good. This procedure was simple, and an ideal result was obtained by the enzyme-linked immunosorbent assay after magnetic separation and coloration of enzyme labelled antibody.

#### Characterization of Fe_3_O_4_@Au-C225 composite targeted MNPs

The Fe_3_O_4_@Au-C225 composite targeted MNPs can be identified by laser particle size analysis, Zeta potential, UV-Vis spectrum, magnetic hysteresis loop and more. The average diameter of Fe_3_O_4_@Au-C225 composite targeted MNPs is 46nm, and shows unimodal and narrow size distribution by laser particle size analysis. Compared to Fe_3_O_4_@Au, which has an average diameter of 35nm, it was slightly increased. This was likely caused by the increase of particle size in the aqueous phase when a protein layer is formed by the adsorption of Fe_3_O_4_@Au surface on C225. The zeta potential value of Fe_3_O_4_@Au-C225 composite targeted MNPs was 11.1±1.8 mV in the neutral environment at a pH of 7.4. This means that C225 was adsorbed on the surface of Fe_3_O_4_@Au composite MNPs. The surface charge was also changed to positive charge. The maximum absorption peak of Fe_3_O_4_@Au composite MNPs at 612nm can be seen in the UV-Vis spectra, whilst the maximum absorption peak of Fe_3_O_4_@Au-C225 composite targeted MNPs was at 630nm. The obvious red-shift showed that antibodies were adsorbed on the surface of gold, a result which was consistent with other studies [29, 30]. The magnetic hysteresis loop of Fe_3_O_4_@Au-C225 composite targeted MNPs was closed to the coincidence S-curve that is similar to Fe_3_O_4_@Au composite MNPs, showing good superparamagnetism, and low remanence and coercivity. The Ms of Fe_3_O_4_@Au-C225 composite targeted MNPs was 51.2emu/g, and was reserved in the Ms of Fe_3_O_4_@Au-C225 composite MNP (51.8emu/g).

### Targeting ability evaluation of Fe_3_O_4_@Au-C225 composite targeted MNPs by MRI

The ideal tracer for displaying microinvasive lesions of tumors should have good resolution, sensitivity, specificity and safety. While the sensitivity of MRI is lower than nuclear medicine imaging, the MRI has a longer effective imaging time window, higher temporal and spatial resolution and better degree of contrast. Thus, MRI shows a wider application foreground in tracing tumor cells *in vivo* [31, 32]. The Fe_3_O_4_@Au-C225 composite targeted MNPs synthesized in our previous study can target EGFR in glioma specifically, and the magnetic property shows super paramagnetic, and a high degree of biocompatibility [3]. So the imaging of glioma cells was taken to approach the effect of MRI on Fe_3_O_4_@Au-C225 composite targeted MNPs *in vivo* and *in vitro* by 7.0 Tesla Micro-MR, and showed the potential of tracing glioma cells *in vivo* by Fe_3_O_4_@Au-C225 composite targeted MNPs.

#### Targeting ability of Fe_3_O_4_@Au-C225 composite targeted MNPs *in vitro*

The superparamagnetism of Fe_3_O_4_@Au-C225 composite targeted MNPs can produce an evident negative contrast effect to reduce target area signal in MR image. The target cell was observed and traced in MR image. The changes in the signals of U251 cells incubated with Fe_3_O_4_@Au-C225 composite targeted MNPs in each sequence of T2*WI, T2WI and T1WI was compared with the agarose control group in the experiment of imaging *in vitro* by MR. The changes in the signal of T2WI and T2*WI were the most obvious, which was consistent with the results of published SPION MRI [33, 34]. The MR imaging of Fe_3_O_4_@Au-C225 composite targeted MNPs made in the laboratory with an equal degree superparamagnetism was explored by learning from the good sensitivity of SPION in T2WI and T2*WI. The specific targeting of Fe_3_O_4_@Au-C225 composite targeted MNPs on glioma cell was evaluated by MR imaging *in vitro*. The results show that the signal intensity of T2WI and T2*WI was decreased after the incubation of U251 cells with Fe_3_O_4_@Au-C225 composite targeted MNPs. The signal intensity in T2WI and T2*WI was decreased minimally after the incubation of U251 cells with Fe_3_O_4_@Au composite MNPs, and Fe_3_O_4_@Au-C225 composite targeted MNPs and C225. This means that Fe_3_O_4_@Au-C225 composite targeted MNPs are effective at targeting U251 cells. The signals decreased notably in T2WI and T2*WI due to more Fe_3_O_4_@Au-C225 composite targeted MNPs entering U251 cell compared to Fe_3_O_4_@Au composite MNPs. This might be a result of the positive charge on the surface of Fe_3_O_4_@Au-C225 composite targeted MNPs, which is able to easily approach the negatively charged membrane and enter cells. C225 mediates the cell endocytosis of composite targeted MNPs. It was also difficult for Fe_3_O_4_@Au composite MNPs to approach and enter U251 cells, which has a negative charge on the surface. The signal intensity of T2WI and T2*WI decreased scarcely after Fe_3_O_4_@Au-C225 composite targeted MNPs were incubated with C225 by adding nutrient medium into U251 cells. This means that Fe_3_O_4_@Au-C225 composite targeted MNPs entering U251 cell was significantly reduced. It is suggested that the receptor-mediated endocytosis of Fe_3_O_4_@Au-C225 composite targeted MNPs is interrupted by dissociative C225, which shows that the entrance of Fe_3_O_4_@Au-C225 composite targeted MNPs into U251 cell was mainly dependent on receptor-mediated endocytosis (Fig 4C).

#### Targeting ability of Fe_3_O_4_@Au-C225 composite targeted MNPs *in vivo*

The transplanted model of human glioma in nude mice made was evaluated on targeting by MRI *in vivo*, in reference to the MR imaging characteristics of Fe_3_O_4_@Au-C225 composite targeted MNPs *in vitro*. The results of MRI *in vivo* show that the intensity and signal change rate of tumor tissues in T2WI and T2*WI at various time points were all decreased after tail vein injection of Fe_3_O_4_@Au-C225 composite targeted MNPs. The most evident difference in statistics can be noted in T2*WI. This may be due to the difference of T2*WI in regards to magnetic susceptibility, where it was more sensitive. The magnetic sensitive effect caused by ferrum was also greater. The signals of stem cell in the kidney and liver, which are labelled with SPIO via renal vein and portal vein, were traced in MRI made by Bos et al. The results show that the decreased effect of imaging signals of T2*WI were the most noticeable, and showed a linear correlation with the dose of iron particles [33]. Kustermann found that the size of the attenuating region of myocardial infarction signals remained consistent in the proton density weighted image and T2*WI image both before and after the injection of labeled cells. However, after the injection of cell labeled iron particles, the size of the proton density weighted image was larger than the T2*WI image. That means T2*WI is more sensitive to SPION, and the target area is increased by the reduced effect of magnetic susceptibility signal [35]. Although the signals of Fe_3_O_4_@Au and Fe_3_O_4_@Au-C225 composite targeted MNPs with C225 were both decreased slightly after the injection at the 2 hour and 8 hour mark, but the decrease was smaller than those of Fe_3_O_4_@Au-C225 composite targeted MNPs and the duration was short. The signal returned to a plain scan level at 24h after the injection of Fe_3_O_4_@Au composite MNPs. By contrast, the signal intensity was still maintained at a lower level at 24h after injection of Fe_3_O_4_@Au-C225 composite targeted MNPs, and showed a decreasing trend with progression of time. This suggest that the content of Fe_3_O_4_@Au-C225 composite targeted MNPs is greater in glioma tissues and is mainly gathered in glioma U251 cells, without the phagocytic degradation by mononuclear phagocyte system via receptor-mediated endocytosis of C225. This is consistent with the results shown in the MRI of U251 cell *in vitro*. Due to the high targeting of tumor cells and obvious imaging effect on T2WI and T2*WI sequences, the Fe_3_O_4_@Au-C225 composite targeted MNPs can be used as MRI negative contrast agents to trace glioma *in vivo*.

## Conclusions

In this study, we discovered that the Fe_3_O_4_@Au-C225 composite targeted MNPs have good superparamagnetism and optical properties. And it could be excellent negative contrast media for MRI. The favorable targeting to glioma *in vitro* and *in vivo* and biocompatibility showed that they have the potential to be used as a tracer for glioma *in vivo*.

## Supporting information

S1 Fig Diameter. The average diameter of Fe3O4@Au-C225 composite targeted MNPs.
(JPG)

S2 Fig Zeta potential report. The Zeta potential value of Fe3O4@Au-C225 composite targeted MNPs.
(GIF)

S1 Table The MRI *in vitro*. The signal intensity in T2WI of U251 cells.
(XLSX)

S2 Table The MRI *in vivo*. The signal intensity and the change rate of tumor.
(XLSX)
