# Supplementary figures and images for: Preparation and characterization of Fe3O4@Au-C225 composite targeted nanoparticles for MRI of human glioma
Source: PLoS One. 2018 Apr 13;13(4):e0195703. doi: 10.1371/journal.pone.0195703 (PMC5898739; doi:10.1371/journal.pone.0195703)

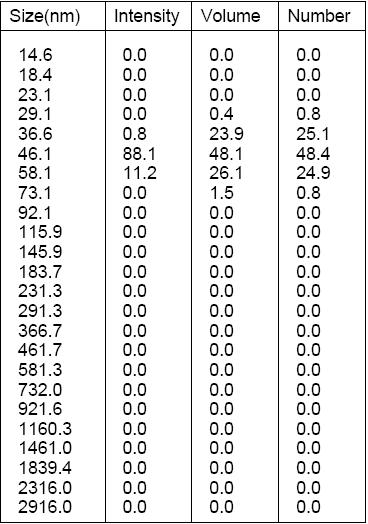

Supplement: S1 Fig — The average diameter of Fe3O4@Au-C225 composite targeted MNPs. (JPG) [file pone.0195703.s001.jpg]

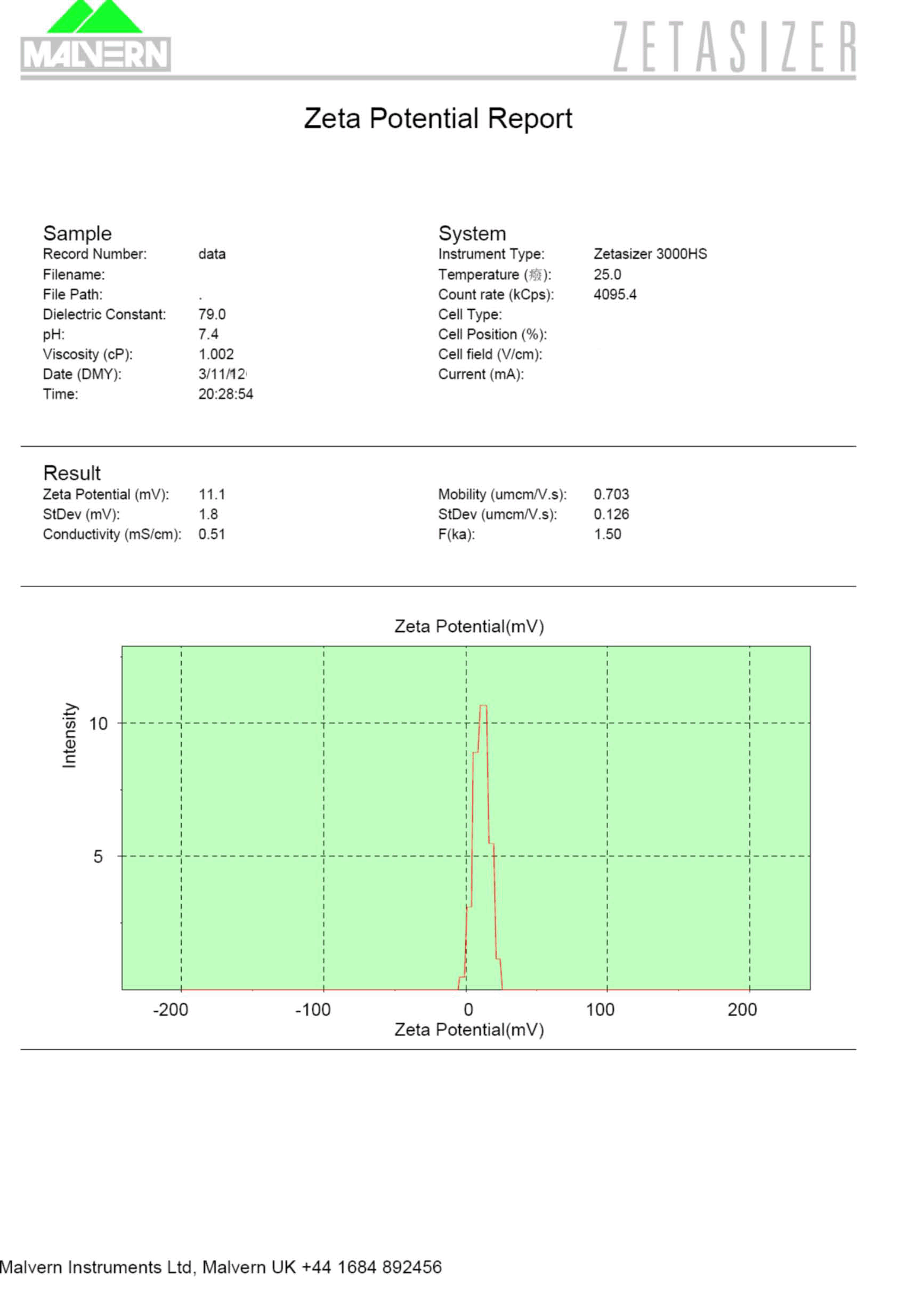

Supplement: S2 Fig — The Zeta potential value of Fe3O4@Au-C225 composite targeted MNPs. (GIF) [file pone.0195703.s002.gif]
